# Identify gestational diabetes mellitus by deep learning model from cell-free DNA at the early gestation stage

**DOI:** 10.1093/bib/bbad492

**Published:** 2024-01-02

**Authors:** Yipeng Wang, Pei Sun, Zicheng Zhao, Yousheng Yan, Wentao Yue, Kai Yang, Ruixia Liu, Hui Huang, Yinan Wang, Yin Chen, Nan Li, Hailong Feng, Jing Li, Yifan Liu, Yujiao Chen, Bairong Shen, Lijian Zhao, Chenghong Yin

**Affiliations:** Beijing Obstetrics and Gynecology Hospital, Capital Medical University, Beijing Maternal and Child Health Care Hospital, Beijing 100026, P. R. China; BGI-Beijing Clinical Laboratories, BGI-Shenzhen, Beijing 101300, P. R. China; Shenzhen Byoryn Technology Co., Ltd., Shenzhen 518118, P. R. China; Shanxi Keda Research Institute, Taiyuan 030000, P. R. China; Beijing Obstetrics and Gynecology Hospital, Capital Medical University, Beijing Maternal and Child Health Care Hospital, Beijing 100026, P. R. China; Beijing Obstetrics and Gynecology Hospital, Capital Medical University, Beijing Maternal and Child Health Care Hospital, Beijing 100026, P. R. China; Beijing Obstetrics and Gynecology Hospital, Capital Medical University, Beijing Maternal and Child Health Care Hospital, Beijing 100026, P. R. China; Beijing Obstetrics and Gynecology Hospital, Capital Medical University, Beijing Maternal and Child Health Care Hospital, Beijing 100026, P. R. China; BGI Genomics, BGI-Shenzhen, Shenzhen 518083, P. R. China; Department of Obstetrics and Gynecology, Peking University Shenzhen Hospital, Shenzhen 518055, P. R. China; Shenzhen Byoryn Technology Co., Ltd., Shenzhen 518118, P. R. China; BGI Genomics, BGI-Shenzhen, Shenzhen 518083, P. R. China; BGI-Beijing Clinical Laboratories, BGI-Shenzhen, Beijing 101300, P. R. China; Shenzhen Byoryn Technology Co., Ltd., Shenzhen 518118, P. R. China; Beijing Obstetrics and Gynecology Hospital, Capital Medical University, Beijing Maternal and Child Health Care Hospital, Beijing 100026, P. R. China; Beijing Obstetrics and Gynecology Hospital, Capital Medical University, Beijing Maternal and Child Health Care Hospital, Beijing 100026, P. R. China; Institutes for Systems Genetics, Frontiers Science Center for Disease-related Molecular Network, West China Hospital, Sichuan University, Sichuan, 610041, P. R. China; BGI Genomics, BGI-Shenzhen, Shenzhen 518083, P. R. China; Beijing Obstetrics and Gynecology Hospital, Capital Medical University, Beijing Maternal and Child Health Care Hospital, Beijing 100026, P. R. China

**Keywords:** gestational diabetes mellitus, deep learning, cell-free DNA, copy number variations

## Abstract

Gestational diabetes mellitus (GDM) is a common complication of pregnancy, which has significant adverse effects on both the mother and fetus. The incidence of GDM is increasing globally, and early diagnosis is critical for timely treatment and reducing the risk of poor pregnancy outcomes. GDM is usually diagnosed and detected after 24 weeks of gestation, while complications due to GDM can occur much earlier. Copy number variations (CNVs) can be a possible biomarker for GDM diagnosis and screening in the early gestation stage. In this study, we proposed a machine-learning method to screen GDM in the early stage of gestation using cell-free DNA (cfDNA) sequencing data from maternal plasma. Five thousand and eighty-five patients from north regions of Mainland China, including 1942 GDM, were recruited. A non-overlapping sliding window method was applied for CNV coverage screening on low-coverage (~0.2×) sequencing data. The CNV coverage was fed to a convolutional neural network with attention architecture for the binary classification. The model achieved a classification accuracy of 88.14%, precision of 84.07%, recall of 93.04%, F1-score of 88.33% and AUC of 96.49%. The model identified 2190 genes associated with GDM, including DEFA1, DEFA3 and DEFB1. The enriched gene ontology (GO) terms and KEGG pathways showed that many identified genes are associated with diabetes-related pathways. Our study demonstrates the feasibility of using cfDNA sequencing data and machine-learning methods for early diagnosis of GDM, which may aid in early intervention and prevention of adverse pregnancy outcomes.

## INTRODUCTION

Gestational diabetes mellitus (GDM) is a condition that affects a significant number of pregnant women. It is characterized by any degree of glucose intolerance that arises during pregnancy [1–4]. According to the International Diabetes Federation, approximately one in six pregnant women experience hyperglycemia, with 84% of these cases being attributed to GDM [4, 5]. GDM tends to affect women at a younger age and has significant adverse effects on both the mother and the fetus [6]. Research has shown that pregnant women who develop GDM are at a higher risk of developing metabolic diseases, such as type II diabetes and obesity, later in life [1, 5]. Furthermore, the adverse intrauterine environment brought about by GDM can permanently damage the fetus’s organ function and structure, resulting in metabolic diseases related to growth and development, such as cardiovascular diseases and diabetes [7–11].

The International Association of Diabetes and Pregnancy Study Group (IADPSG) recommended diagnostic criteria based on the study results of the Hyperglycemia and Adverse Pregnancy Outcome (HAPO) [5]. However, there is no consensus on the diagnosis criteria for early GDM due to insufficient data in diagnosing and testing GDM in early gestation [12, 13]. The delay in diagnosis and the subsequent disorder management increases the risk of poor pregnancy outcomes, especially for high-risk women with advanced age or obesity [14–17]. For example, studies suggest that fetal macrosomia, a prevalent complication associated with GDM, may develop as early as the 20th week of pregnancy [18, 19].

Copy number variations (CNVs) can be a possible biomarker for GDM diagnosis in the early gestation stage. Former studies have reported CNVs related to pregnancy complications by sequencing the cell-free DNA (cfDNA) in the maternal plasma [20, 21]. A study with cfDNA had identified CNVs in three chromosome regions related to a high risk of GDM. Gene enrichment analysis reveals that the genes in these regions contain some alpha- and beta-defensin family members, such as DEFA1, DEFA3 and DEFB1, copy number variations of which are associated with type I and II diabetes [22]. However, there are no existing methods for early prediction of GDM based on the CNV markers.

This study proposes a machine-learning method for early-stage GDM prediction using low-coverage genome-wide sequencing data of cfDNA in maternal plasma. A non-overlapping sliding window method with matrix imputation was applied for CNV coverage screening, and a deep neural network with attention architecture was used for binary classification. The model achieved a classification accuracy of 88.14%, precision of 84.07%, recall of 93.04%, F1-score of 88.33% and AUC of 96.49%. We also identified 2190 genes associated with GDM in the early gestation stage, including DEFA1, DEFA3 and DEFB1. These genes were enriched in 327 GO terms and 18 KEGG pathways with an adjusted *P*-value lower than 0.05. Many of them have previously been associated with diabetes mellitus, including the glutamate receptor signaling pathway [23, 24] and the type II diabetes mellitus pathway [25, 26]. The study’s findings suggest that the proposed machine-learning method can effectively identify early-stage GDM biomarkers and provide a basis for early diagnosis and management of the condition.

## MATERIALS AND METHODS

### Sample collection and clinical PCA

A total of 5085 pregnant women from north regions of Mainland China were retrospectively enrolled in this study, and blood samples were collected between 12 and 26 weeks after gestation. The clinical information pertaining to age and weight was collected and is listed in Supplementary Table 1. The majority of the samples (62.20%) were collected between 12 and 18 weeks after gestation. The diagnosis of gestational diabetes mellitus (GDM) followed the recommended criteria by the IADPSG. Pregnant women underwent a 75 g oral glucose tolerance test between 24 and 28 weeks of gestation. GDM was diagnosed if any of the following glucose values was equal to or exceeded: fasting plasma glucose of 5.1 mmol/L, 1-h plasma glucose of 10.0 mmol/L or 2-h plasma glucose of 8.5 mmol/L. Accordingly, 1942 samples who were diagnosed as GDM based on these diagnostic criteria were enrolled. In the subsequent analysis, the normal samples were labeled as 0, while the GDM samples were assigned as 1. Principal component analysis (PCA) was conducted using the clinical attributions as features. Samples with missing values were filtered out, and the values of remaining sample features were normalized to a range of −1 to 1. The PCA was performed using the ‘scikit-learn’ package in Python. The study was approved by the Hospital Ethics Committee of the Beijing Obstetrics and Gynecology Hospital (approval no. 2018-KY-003-02), and all patients provided signed informed consent. The experiments were conducted in accordance with the guidelines of the National Heath Commission of the People’s Republic of China.

### DNA extraction, library construction and genome sequencing

Five milliliters of peripheral venous blood samples was collected into tubes containing ethylene diamine tetraacetic acid from each participant. All blood samples were processed within 8 h by a double-centrifugation protocol. Blood samples were first centrifuged at 1600 × *g* for 10 min, and the supernatant was recentrifugated at 16 000 × *g* for 10 min to remove residual cells. The resultant cell-free plasma was stored at −80°C. Then, 200 μL of maternal plasma was used for cfDNA extraction by BGISP-300 (BGI, Shenzhen, China) with a nucleic acid extraction kit (BGI, Shenzhen, China).

After DNA extraction, end-repair was carried out by adding end-repair enzymes under the following cycle conditions: 37°C for 10 min and 65°C for 15 min, followed by adaptor ligation with label-adaptor and ligase at 23°C for 20 min. After the end-repair and adaptor ligation, PCR was performed to amplify DNA to the desired concentration under the following cycle conditions: 98°C for 2 min, then 12 cycles at 98°C for 15 s, 56°C for 15 s and 72°C for 30 s, with a final extension at 72°C for 5 min. The DNA amplification products were quantified by Qubit^®^ 2.0 (Life Tech, Invitrogen, Carlsbad, CA, USA) using Qubit^TM^ dsDNA HS Assay Kits (Life Tech, Invitrogen, USA), and the concentration ≥2 ng/μL was regarded as qualified standards. The volume was calculated according to the concentration of each sample, and each sample of the same mass was mixed by pooling. The fetal chromosome aneuploidies (T21, T18 and T13) detection kit (Combinatorial Probe-Anchor Synthesis Sequencing Method, CPAS) (BGI, Shenzhen, China) was used for library construction.

Double-strand DNA was thermally denatured into single-strand DNA after pooling, followed by the addition of cyclic buffer and ligase to create DNA circles by cyclization. Qualified DNA circles were used to make DNA Nanoballs (DNBs) by rolling-circle replication. The concentration of DNBs was quantified by Qubit^®^ 2.0 using Qubit^TM^ ssDNA assay kits (Life Tech, Invitrogen, USA), and DNB concentrations within the range of 8–40 ng/μL were considered appropriate. Then, DNBs were loaded onto chips and sequenced on a BGISEQ-500 sequencing platform (BGI, Shenzhen, China) by the SE35 model.

The average depth of sequencing for each sample was approximately 0.2×, with a minimal number of unique sequencing reads of no less than 6 million obtained per sample for analysis.

### Sequencing alignment and quality control

**Figure 1:**
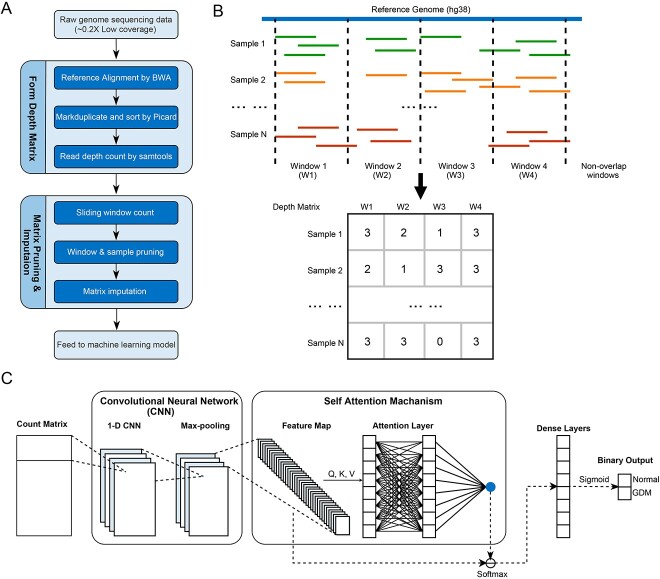
An overview of the GDM classification model. (**A**) The pipeline for processing sequencing data to generate count matrix for deep learning model. (**B**) An example for the count matrix constructure. (**C**) The deep learning architecture of our model.

The sequencing data processing steps are shown in Figure 1A. We first aligned the genome sequencing data of cfDNA to the human genome (hg38) by bwa-mem2 (https://github.com/bwa-mem2/bwa-mem2) [27]. Due to the low sequencing coverage, we filtered out the reads that are not in USCS Mappability track wgEncodeCrgMapabilityAlign100mer.bigWig (https://genome-euro.ucsc.edu/cgi-bin/hgTrackUi?db=hg38&g=mappability). The UCSC Mappability track provides information about the mappability of genomic regions. It indicates the regions in the genome that can be accurately mapped or aligned by sequencing reads. To estimate the impact of complex genome regions like segmental duplications, we further procured the segmental duplicated regions dataset from the UCSC Genome Browser’s repository and embarked on an in-depth analysis. This analysis encompassed an evaluation of the distribution of base pair sequencing depths within both segmental duplication and non-segmental duplication regions, as showcased in Supplementary Figure 3. Specifically, we can observe that base pairs situated within segmental duplicated regions tend to exhibit diminished read depth. This suggests that these regions encounter challenges with respect to read mapping, likely attributed to their intricate structural characteristics. Thus, we excluded these regions from our subsequent analyses to ensure the reliability of our conclusions. After removing duplicated reads by Picard (version 2.27.5) [28], samtools (version 1.9) [29] was used to calculate the sequencing coverage along the reference genome for each sample. The average sequencing coverage among the samples in this study was 0.19×. We filtered out the samples with average coverage lower than 0.15×. We comprehensively evaluated quality control (QC) factors, including GC content, alignment rate, repeat rate and Q20, on the aligned data. The specific QC statistics for each sample can be found in Supplementary Table 1. A PCA was employed to assess the impact of QC statistics on sample differentiation within both the normal and GDM groups. This analysis aimed to verify that the presence of QC statistics did not introduce any confounding effects when distinguishing between these variant groups.

### Bias correction and coverage count

The genome reference was further divided by a non-overlap sliding window method with different window sizes. The window sizes were set to 50, 20, 10 and 5 k separately. Preceding the read counting process, a bias correction procedure was implemented using HMMcopy. This correction was applied to mitigate any potential influence stemming from variations in GC content and mappability, both of which could affect read depth. To visually demonstrate the impact of GC content and mappability on read depth, scatter plots were generated employing varying window sizes. Each data point within these plots corresponds to a sliding window, with the respective GC content (Supplementary Figure 2) or mappability values (Supplementary Figure 3) plotted on the *x*-axis and the read depth values on the *y*-axis. The mappability data source was acquired from the National Center of Biotechnology Information (NCBI) mappability track of hg38 (accessible via the website). Subsequent to bias normalization, it was observed that GC content and mappability displayed similar distributions in both the normal and GDM groups. This concurrence in distribution emphasizes that the observed variability in read depth between the two groups is not primarily driven by differences in GC content or mappability. We counted the coverages along the reference for each window size by summing up the coverages of base pairs inside the windows. We further visualized the scaled average coverage of normal samples, GDM samples and their diversity by scSVAS [30].

### Feature selection, window pruning and coverage matrix imputation

We formed the window coverage matrix as shown in Figure 1B, with each row representing a sample and each column representing a window. The elements in the matrix are the window coverages. We normalized the elements with average coverages of samples row by row. To reduce the model complexity in the downstream binary classification task, we pruned out the windows by the following rules: (1) windows with over 80% samples with normalized coverage lower than 0.1 were dropped out; (2) windows with similar average coverages in normal and GDM samples (the difference is less than 0.01) were dropped out. To avoid the impact of read dropout (regions that cannot be sequenced) and sequencing errors due to low sequencing coverage, we further performed matrix imputation by SCOIT (https://github.com/deepomicslab/SCOIT) [31]. SCOIT has been proven effective in single-cell data and can also be used for value imputation in matrix with other data sources. The imputed matrix was used for subsequential machine-learning analysis.

### Deep learning model and parameter optimization

Our model was designed to identify GDM cases in the early gestation stage based on low-coverage sequencing data of cfDNA. The coverage matrix generated by the non-overlapping sliding window was the input of the model. We built a CNN-based deep neural network with a self-attention layer to achieve binary classification, as shown in Figure 1C. In detail, the input layer passes the matrix to two 1D convolution layers, with each convolution layer followed by a maximum pooling layer. After the convolution layers, we built a self-attention layer. A self-attention layer was joined to link the last convolution pooling layer and dense layers. Two fully connected dense layers with a dropout of 0.4 were then connected to the attention layer. Sigmoid was used as the activation function of the output layer to indicate whether the input sample was from a GDM patient. The deep learning model was constructed by the Keras package of Tensorflow [32]. The model’s hyperparameters, along with their corresponding explanations, are presented in Supplementary Table 4. To optimize these hyperparameters, we conducted a grid search, systematically exploring different combinations within specified upper and lower bounds. The bounds and search step for the hyperparameters are also detailed in Supplementary Table 4.

### Training and evaluation

The dataset was randomly split into a training dataset (75%), a validation dataset (5%) and a testing dataset (20%). The model was trained using random weight initialization with a batch size 512 for 100 epochs. The Adam method was applied in the optimization process with 1e−5 as the learning rate and binary cross-entropy as the loss function. We adopted the dropout strategy to prevent overfitting. To evaluate the stability of our model, we conducted a 10-fold cross-validation on the dataset. During this process, we set the classification threshold to 0.5 by default, indicating that any prediction above this threshold was considered GDM.

### Benchmarking settings

We compared the performance of our model on our dataset with other machine-learning models, including the Random Forest (RF) model and the Support Vector Machine (SVM) model. The models were implemented by the Python scikit-learn package (version 1.20) [33]. The default parameters were adopted in the two models. We conducted a comparative analysis between our method and a methodology involving the identification of CNVs followed by the diagnosis of GDM by detecting pathogenic CNVs. To achieve this, we employed ichorCNA [34], a robust tool designed for estimating the tumor fraction in cell-free DNA through ultra-low-pass whole genome sequencing (ULP-WGS) at 0.1× coverage. Due to the absence of a normal panel, we performed CNV calling using window sizes of 10, 50, 500 and 1000 k, for which ichorCNA provides corresponding databases (gcWig file and mapWig file). The command-line executions for readCounter and ichorCNA followed the default parameters as outlined in the ichorCNA usage manual.

### Performance evaluation

We used the confusion matrix by the validation dataset to measure the performance of the classification model. For a binary classification model, the confusion matrix is a 2 $\times$ 2 matrix with the ratio of true-positive cases, true-negative cases, false-positive cases and false-negative cases. We further calculated the accuracy (the percentage of samples that are correctly predicted), precision (the percentage of predicted positive cases that are correctly predicted), recall (the percentage of actual positive cases that are correctly predicted) and F1-score (a harmonic means of precision and recall) from the confusion matrix. We demonstrated the performance of our classification model at all classification thresholds by the receiver operating characteristic (ROC) curve. We calculated the area under the ROC curve (AUC) to provide an aggregated measure of the model performance across all possible classification thresholds. We also applied direct bootstrapping by random sampling to estimate the uncertainty of the model. We performed 10 000 experiments for each window size with bootstrap sampling and calculated the average AUC with a confidential interval. For each experiment, we also measured the correlation coefficient to determine whether the model-predicted value has a relationship with the true label. The *P*-value was calculated by the ‘pearsonr’ function in the Python package ‘scipy’.

### Finding significant regions by self-attention layer

Our model trained the weights of the windows in the self-attention layer. The window weight indicates the positive contribution of the window in the binary classification of GDM. The model generated a 1 × *N* weight array in the training process, where *N* is the number of windows. We sort the windows by their weights to obtain the top 5000 windows with the most significant discrimination power in the binary classification and mark the regions on the reference indicated by the windows.

### Gene annotation and enrichment

The selected cfDNA window regions were annotated to genes on Ensembl release 108. Then, we performed the Gene Ontology (GO) terms and Kyoto Encyclopedia of Genes and Genomes (KEGG) pathway enrichment analyses using the R package ‘clusterProfiler’. Benjamini–Hochberg correction (FDR) was used for adjusted *P*-value calculation. Adjusted *P*-value less than 0.05 was considered significant.

## RESULTS

### Similar clinical characteristics of normal and GDM groups

**Figure 2:**
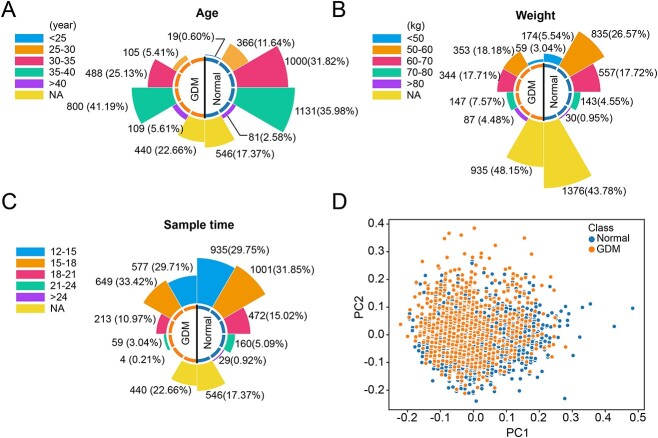
Clinical information of the collected samples. (**A**) The age distribution of the normal group and GDM group. (**B**) The weight distribution of the normal group and GDM group. (**C**) The sample time distribution of the normal and GDM group. (**D**) PCA scatter plot of normal samples and GDM samples.

Of the 5085 pregnant women, 1942 were diagnosed with GDM. The rose diagrams showed that the distributions of samples across all three clinical attributes, i.e. age, weight and sample time, were similar in the normal and GDM groups (Figure 2A–C). More than half of the samples were obtained from patients over 35 years old in normal and GDM groups. The GDM group had a higher proportion of patients with advanced maternal age compared to the normal group. Among the clinical attributes, patient weight had the most missing values. Most patients had a weight range between 50 and 70 kg for known values. Regarding the sample time, the majority of samples were collected between 12 and 18 weeks after gestation in both the normal (61.60%) and GDM (62.10%) groups. Given the common perception that advanced maternal age and higher body weight predispose pregnant women to a greater risk of developing GDM, our objective in conducting the PCA was to confirm the absence of any bias related to clinical information within our dataset. The PCA results revealed a mingling of samples from both groups, signifying that the two groups exhibited comparable clinical characteristics (Figure 2D).

### Copy number diversity on different window size

**Figure 3:**
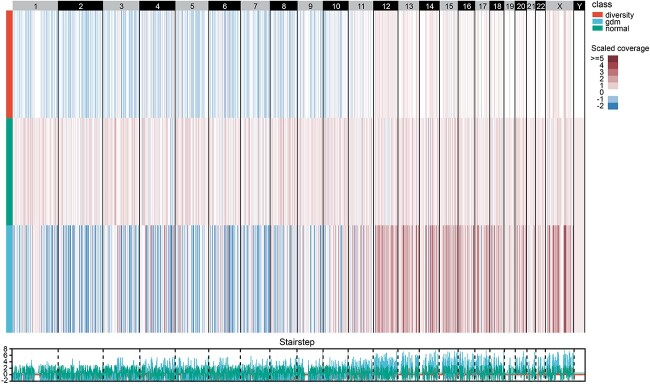
Copy number diversity on different window size along the whole chromosome.

After generating the coverage matrix with the number of aligned reads in each window, we performed sample filtering and window pruning to the coverage matrix. Six hundred fifteen samples (194 GDM samples and 421 normal samples) were filtered out as their sequencing coverage was lower than 0.15×. The non-overlapping sliding window method generated 617 689, 308 861, 154 447 and 61 799 windows initially for the window sizes 5, 10, 20 and 50 k. After the window pruning process followed the rules mentioned in the method, 20 637, 11 288, 4520 and 2114 windows were left separately for different window sizes. The processed coverage matrixes were then fed to perform matrix imputation and binary classification. Figure 3 demonstrated the normalized diversity heatmap of the coverage diversities between GDM and normal samples at the whole genome sequencing level with variant window size. GDM groups showed a greater magnitude of copy number alterations compared to the normal group. Specifically, more copy number loss events on chromosomes 1–9 and more copy number gain events on chromosomes 12–19, 20 and X were observed in GDM groups.

### Optimal window size and the performance of the model

**Figure 4:**
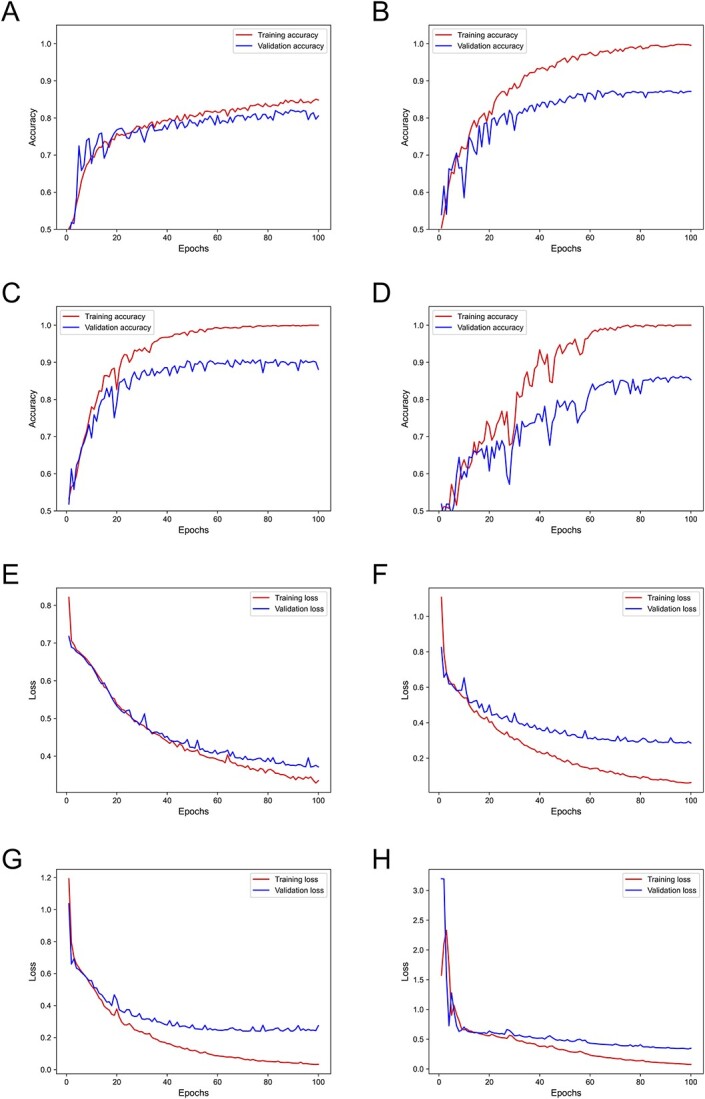
Model learning performance on different window size. (**A**–**D**) Learning accuracy curve for window size 50, 20, 10 and 5 k, respectively. (**E**–**H**) Learning loss curve for window size 50, 20, 10 and 5 k, respectively.

The training model utilized 3353 (75%) samples (1326 GDM samples and 2027 normal samples) for training, 223 (5%) samples (80 GDM samples and 143 normal samples) for validation and 894 (20%) samples (342 GDM samples and 552 normal samples) for testing. The learning and loss curves for different window sizes are shown in Figure 4. We can observe that the model performance is relevant to the window size as smaller windows provide higher resolution in screening CNV. As illustrated in Figure 4A–D, the model started to converge at around epochs 10–40. When the window size is 10 k, the model provided the best validation accuracy, around 86%. The model tended to have overfitting with the dataset generated by window size 5 k as smaller window sizes introduced more candidate windows as features. The training loss shown in Figure 4E–H has similar results with learning curves.

**Figure 5:**
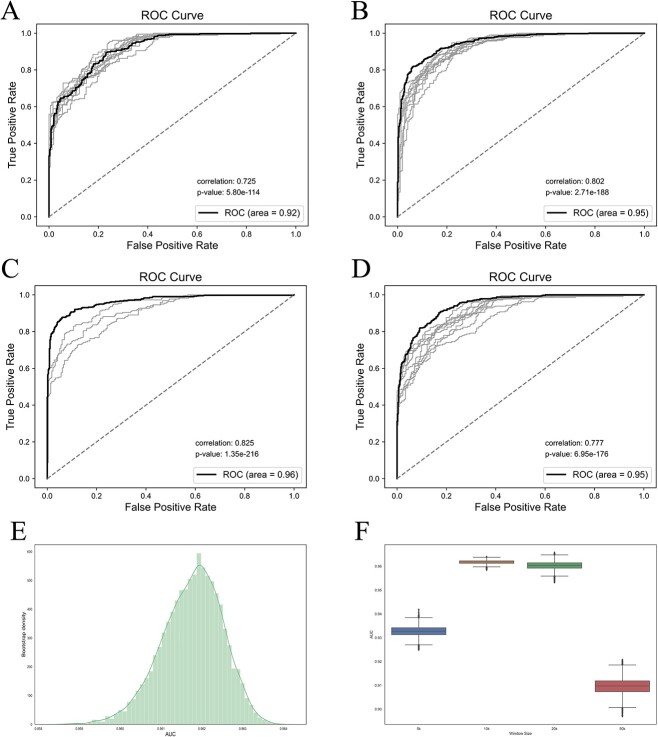
Model performance on test dataset. (**A**–**D**) ROC curve for window size 50, 20, 10 and 5 k, respectively. The black line represents the ROC curve generated by the test dataset, while the gray lines represent the ROCs obtained from cross-validation. (**E**) The AUC distribution of 10 k window size on 10 000 bootstrapping experiments. (**F**) The AUCs of variant window sizes on bootstrapping experiments.

To assess the stability of our model, we conducted 10-fold cross-validation experiments using different window sizes. The results are presented in Figure 5A–D, which showcases the ROC curves along with the corresponding AUC values. In each subfigure, the black line represents the ROC curve generated by the test dataset, while the gray lines represent the ROCs obtained from cross-validation. We also annotated the correlation and *P*-values between the model’s predictions and the true values in the test dataset for each subfigure. Notably, all *P*-values were below 0.05, indicating a strong and statistically significant correlation between the predictions and the true values. Furthermore, the ROC curves align consistently with the learning curve derived from the validation data, demonstrating that our model achieves optimal performance when the window size is set to 10 k. The model performed best when the window size is 10 k with an accuracy of 88.14%, sensitivity of 84.07%, recall of 93.04%, F1-score of 88.33% and AUC of 96.49%. Table 1 shows the detailed confusion matrix and assessment metrics.

**Table 1:** Details on confusion matrix and performance measurement metrics of three machine-learning models with different window sizes

Window size	50 k			20 k			10 k			5 k		
Performance metrics	SVM	RF	DL	SVM	RF	DL	SVM	RF	DL	SVM	RF	DL
Accuracy	80.43%	81.10%	80.54%	81.88%	73.83%	87.14%	84.12%	72.60%	88.14%	81.99%	75.62%	85.35%
Precision	79.67%	84.92%	82.16%	79.66%	76.92%	88.50%	82.97%	77.26%	84.07%	81.73%	78.47%	80.01%
Recall	79.11%	72.55%	76.05%	80.84%	58.23%	86.81%	78.35%	54.04%	93.04%	78.35%	57.49%	92.81%
F1	79.39%	78.25%	78.99%	80.24%	66.28%	87.65%	80.01%	63.59%	88.33%	80.01%	66.36%	85.93%
AUC	90.07%	90.62%	91.64%	91.00%	83.51%	95.03%	94.29%	82.62%	96.49%	91.24%	84.71%	94.58%
Acc std on cross val.	0.019	0.007	0.026	0.010	0.012	0.031	0.019	0.007	0.022	0.047	0.018	0.099
Prediction Correlation	0.762	0.704	0.725	0.730	0.601	0.802	0.787	0.580	0.825	0.736	0.603	0.777
P-value	1.10E−153	1.88E−121	5.80E−114	1.54E−120	1.59E−93	2.71E−188	9.28E−138	4.89E−89	1.35E−216	1.03E−124	6.81E−98	6.95E−176

DL: our Deep Learning model.

We also performed bootstrapping to evaluate the robustness of the model with 10 000 experiments on each window size. Figure 5E and F demonstrated the performance of our model with testing data random sampling. The boxplot in Figure 5E shows the AUCs at variant window sizes. The mean AUCs for window sizes 50, 20, 10 and 5 k are 0.92, 0.94, 0.96 and 0.93, respectively. The window size of 10 k provided the best AUC and smallest variation among the experiments. Figure 5F shows the distribution of AUCs with window size 10 k among the experiments. The density forms a normal distribution with [0.952, 0.968] as a 95% confidential interval.

We also compared the performance of our model with machine-learning models, including SVM and RF, as shown in Table 1. All of the results are statistically significant (*P*-value ≤ 0.05). The 10 k window size provided the best performance on the testing dataset in the two comparison methods. Our model achieved the best classification performance in terms of AUC and accuracy among the three methods.

We also compared our method with methodology of involving the identification of CNVs. We have incorporated the experiments involving ichorCNA with CNV screening window size 10, 50, 500 and 1000 k. We conducted separate screenings for each of these window sizes. Regrettably, when utilizing a 10 k window size, ichorCNA encountered an error and failed to produce valid outcomes, consistently displaying the error message ‘zero-width neighborhood. make span bigger.’ across all samples. In contrast, for other window sizes, ichorCNA yielded meaningful integer-based CNV results.

Before applying our deep learning model to the dataset, we executed a feature pruning procedure to reduce the number of CNV windows. This process adhered to two criteria: (1) the exclusion of windows where over 90% of samples (across both normal and GDM groups) shared the same CNV value; and (2) the elimination of windows where the disparity in average CNV values between the normal and GDM groups was below 0.1. This culling procedure led to the retention of 3846, 595 and 8 valid windows for the 50, 500 and 1000 k window sizes, respectively. The 1000 k window size was subsequently disregarded due to an insufficient number of viable windows, while the 50 and 500 k sizes proceeded to be utilized for our DL model. Supplementary Table 5 presents the performance metrics of the performance for DL model with ichorCNA called CNV dataset, and Supplementary Figure 3 portrays the learning curve. The window size 50 k provided the best results, with accuracy 69.84%, precision 67.69%, recall 72.13%, F1-score 69.84% and AUC score 79.89%.

### cfDNA fragments covered diabetes-associated genes

We joined a self-attention layer in the CNN-based model to measure the importance of the input windows. The self-attention layer generated a weight array indicating the corresponding window’s discrimination power in the binary classification of GDM. Here, we utilized the results obtained by window size 10 k as it provided the best performance. We sorted the windows by weight and extracted 5000 windows for further analysis. As shown in Figure 3, the observed differences in coverage highlight the potential utility of these regions for accurate classification and further emphasize their relevance in understanding the characteristics associated with GDM. We annotated 2190 genes in the window regions (Supplementary Table 2). The previously discovered alpha- and beta-defensin genes, DEFA1, DEFA3 and DEFB1 [22], also occurred in our gene list.

**Figure 6:**
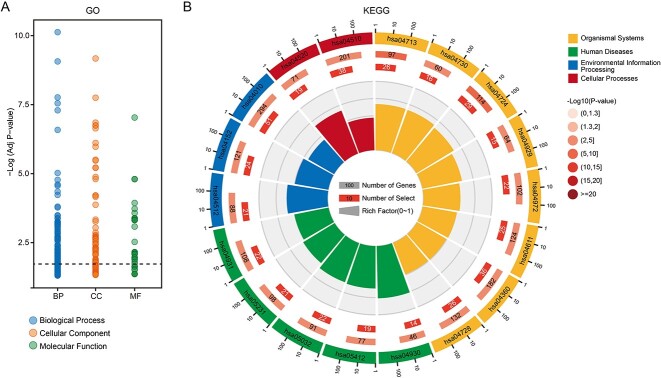
The results of GO term and KEGG pathway enrichment of annotated genes. (**A**) Enriched GO terms with adjusted *P*-value smaller than 0.05. (**B**) Enriched KEGG pathways with adjusted *P*-value smaller than 0.05.

We performed GO and KEGG enrichment analysis on the annotated genes. The genes covered by cfDNA fragments were enriched in 327 GO terms and 18 KEGG pathways (*P*_adj_ < 0.05; Figure 6, Tables 2–3 and Supplementary Table 3). Among GO terms, glutamate (GO:0007215; adjusted *P*-value = 2.34e−5) and ionotropic glutamate receptor signaling pathways (GO:0035235; adjusted *P*-value = 1.52e−5) were associated with type I and type II diabetes [23, 24]. Studies have shown that diabetes partially loses its glucoregulatory mechanism due to Ca^2+^ channel activity and gene expression decrease in alpha cells [35, 36]. In addition, a complete glucagon response to hypoglycemia relies on positive autocrine feedback mediated by extracellular glutamate, which acts on ionotropic glutamate receptors of the AMPA/kainite type [37]. Besides, forebrain development (GO:0030900; adjusted *P*-value = 4.84e−7) and forebrain cell migration (GO:0021885; adjusted *P*-value = 2.92e−4) are descendant nodes of brain development in GO Term Tree. The changes in brain signaling systems have been reported to be essential in the etiology and pathogenesis of type II diabetes mellitus and metabolic syndrome [38]. GTPase regulator activity (GO:0030695; adjusted *P*-value = 3.55e−8) participated in post-translational modifications that were also linked to diabetes mellitus [39]. Genes were enriched in the type II diabetes mellitus pathway (hsa04930; adjusted *P*-value = 1.76e−4), consistent with the reported correlation between type II diabetes mellitus and PDM [25, 26]. Consistent with the GO term enrichment analysis, we can observe a glutamatergic synapse pathway (hsa04724; adjusted *P*-value = 8.07e−4) that consists of glutamate localization inside presynaptic vesicles and glutamate receptors on the postsynaptic membrane. Other enriched KEGG pathways that are known related to type II diabetes mellitus include adherens junction (hsa04520, adjusted *P*-value = 8.82e−3), MAPK signaling pathway (hsa04010; adjusted *P*-value = 8.82e−3), pancreatic secretion AMPK signaling pathway (hsa04972; adjusted *P*-value = 2.13e−3), platelet activation (hsa04611; adjusted *P*-value = 2.54e−2), AMPK signaling pathway (hsa04152; *P*-value = 3.28–2) and insulin resistance (hsa04931; adjusted *P*-value = 3.33–2) [40–42]. The adherens junction plays a role in the pathogenesis of type II diabetes mellitus through repercussion for the endocrine pancreas, intestinal barrier and kidney dysfunctions [40].

**Table 2:** List of top 10 GO terms at each oncology

ID	GeneRatio	BgRatio	p.adjust
hsa04724	29/865	114/8216	0.00080703
hsa04713	26/865	97/8216	0.00080703
hsa04520	19/865	71/8216	0.008818431
hsa04360	36/865	182/8216	0.008818431
hsa05032	22/865	91/8216	0.008818431
hsa04930	14/865	46/8216	0.008818431
hsa04010	51/865	294/8216	0.008818431
hsa04510	38/865	201/8216	0.008818431
hsa04512	21/865	88/8216	0.008818431
hsa05412	19/865	77/8216	0.009812068
hsa04730	16/865	60/8216	0.01031654
hsa04972	22/865	102/8216	0.021381667
hsa04611	25/865	124/8216	0.025380953
hsa05231	21/865	98/8216	0.025380953
hsa04728	26/865	132/8216	0.025380953
hsa04152	24/865	121/8216	0.032835659
hsa04931	22/865	108/8216	0.033347701
hsa04929	15/865	64/8216	0.040114895

**Table 3:** List of enriched KEGG pathways

ONTOLOGY	Description	GeneRatio	BgRatio	p.adjust
BP	Synapse organization	100/1856	432/18903	9.86E−13
BP	Cell junction assembly	94/1856	430/18903	1.70E−10
BP	Regulation of neuron projection development	91/1856	446/18903	1.76E−08
BP	Dendrite development	58/1856	237/18903	5.55E−08
BP	Axon development	95/1856	490/18903	7.32E−08
BP	Axonogenesis	84/1856	438/18903	1.37E−06
BP	Positive regulation of cell projection organization	70/1856	352/18903	5.90E−06
BP	Synapse assembly	44/1856	184/18903	1.32E−05
BP	Regulation of neuron migration	19/1856	47/18903	1.99E−05
BP	Regulation of axonogenesis	38/1856	156/18903	5.78E−05
CC	Synaptic membrane	83/1938	378/19869	6.95E−10
CC	Postsynaptic membrane	61/1938	269/19869	9.15E−08
CC	Glutamatergic synapse	68/1938	324/19869	2.03E−07
CC	Adherens junction	45/1938	179/19869	3.34E−07
CC	Neuron to neuron synapse	69/1938	351/19869	1.34E−06
CC	Ion channel complex	61/1938	296/19869	1.34E−06
CC	Cation channel complex	50/1938	222/19869	1.34E−06
CC	Sarcolemma	35/1938	136/19869	5.43E−06
CC	Cell leading edge	76/1938	421/19869	7.19E−06
CC	Asymmetric synapse	62/1938	327/19869	1.69E−05
MF	Actin binding	90/1880	439/18432	7.49E−08
MF	GTPase regulator activity	89/1880	488/18432	1.32E−05
MF	Nucleoside-triphosphatase regulator activity	89/1880	488/18432	1.32E−05
MF	Actin filament binding	47/1880	219/18432	0.00016932
MF	Protein serine/threonine kinase activity	76/1880	430/18432	0.000275814
MF	Protein serine kinase activity	66/1880	363/18432	0.000422209
MF	Beta-catenin binding	24/1880	87/18432	0.000455544
MF	Calmodulin binding	42/1880	200/18432	0.000455544
MF	Gated channel activity	62/1880	341/18432	0.000455544
MF	Ionotropic glutamate receptor activity	10/1880	19/18432	0.000455544

## DISCUSSION

GDM is a prevalent pregnancy complication that can have detrimental effects on maternal and fetal health. However, GDM is usually diagnosed after 24 weeks of gestation, which is a delayed detection. Therefore, it is necessary to develop consensus diagnostic criteria for GDM in the early gestational stage. To address this issue, this study proposed a convolutional neural network (CNN)-based deep learning model that uses low-coverage sequencing data of cell-free DNA (cfDNA) samples to classify GDM in the gestation stage as early as 12 weeks of gestation. To construct our proposed GDM classification model, CNVs needed to be called from the aligned data. For this purpose, we employed the widely recognized CNV calling software tailored for low-depth data, ichorCNA. The obtained count of valid windows was limited for all selected window sizes. The window size of 50 k yielded the most promising outcomes through our deep learning model, boasting an accuracy of 69.84%, precision of 67.69%, recall of 72.13%, F1-score of 69.84% and an AUC score of 79.89%. Subsequently, we adopted a non-overlapping sliding window methodology that aggregated the coverages of base pairs within each window from the aligned data. This procedure generated a window coverage matrix, which served as the input for our CNN-based deep neural network. The DL model achieved high accuracy, with an AUC, accuracy, precision, recall and F1-score of 96.49, 88.14, 84.07, 93.04 and 88.33%, respectively. The proposed model demonstrated high stability, evidenced by the mean AUC of 0.96 from bootstrapping with 10 000 experiments. Compared to identifying CNVs from extremely low-coverage samples, our method allows us to achieve higher-resolution results in cases where non-integer CNV signals are present within the CNV segments, leading to enhanced sensitivity and accuracy in distinguishing between normal and GDM samples. We suggest that the proposed model can be useful in the clinical screening of GDM, potentially leading to better health outcomes for both mothers and fetuses.

It is crucial to consider interpreting the biological mechanism underlying our model. Previous research has shed light on the segmental nature of cfDNA footprints, which exhibits sequence segment bias toward genome regions. The relatively minor diversity observed in both the GDM and normal groups could potentially be attributed to this segmental DNA originating from maternal conditions. To delve deeper, we assessed the discrimination scores of specific genomic windows. These scores serve as indicators of the model’s ability to distinguish between GDM and normal patients. By focusing on regions that are either frequently present or notably absent in GDM patients, we can increase the likelihood of identifying biologically significant regions. Furthermore, our analysis included enrichment studies, as well as an exploration of GO terms and KEGG pathways associated with these highlighted regions. These steps are essential for understanding the functional implications of the identified regions and their potential roles in the context of gestational diabetes mellitus (GDM). We believe that our proposed model can be easily adapted to other diseases with low-coverage sequencing data.

Despite demonstrating high accuracy in classifying GDM in the early gestation stage, the study has limitations. The model was tested on our dataset, and more data from other sources and variant demographic groups following a similar sequencing protocol to our dataset should be included to strengthen the credibility of the model. In addition, the CNV genes identified in the study were not verified experimentally due to the difficulty in the follow-up of GDM cases. Further studies are required to validate the identified genes through experimental verification. Regions frequently present or absent in GDM patients provide higher discrimination power in the binary classification task and, thereby, are more likely to be identified. It is important to acknowledge that the validation of these findings necessitates high-coverage sequencing data derived from maternal cfDNA. This validation process is a critical next step in our research but may require further efforts in future work to obtain the required data for a more comprehensive and conclusive assessment.

Nonetheless, the study highlights the effectiveness of deep learning and convolutional neural networks in scanning CNVs with low-coverage sequencing data for GDM classification in the early gestation stage.

**Key Points**

- We proposed a convolutional neural network (CNN) model for classifying gestational diabetes mellitus (GDM) from 5085 individuals with low-coverage cfDNA sequencing data in early gestation stage. Among the recruited individuals, 1942 were diagnosed as GDM. The model achieved an accuracy of 88.14%, precision of 84.07%, recall of 93.04%, F1-score of 88.33% and AUC of 96.49%.
- Sequencing coverage matrix generated by non-overlapping sliding window screening provide candidate copy number variation (CNV) regions in low-coverage sequencing data.
- Self-attention architecture revealed potential GDM-associated genes and CNV regions. Gene Oncology (GO) term and KEGG enrichment analysis on the potential genes identified diabetes-related pathways.
- Our machine-learning model showed high efficiency on predicting GDM in early pregnancy stage, which may aid in early intervention of GDM and prevention of adverse pregnancy outcomes.

## Supplementary Material

Supplementary_material_bbad492

## Author Biographies

**Yipeng Wang**, PhD, is a research fellow in Prenatal Diagnostic Center of Beijing Obstetrics and Gynecology Hospital, Capital Medical University. His research focuses on genetic clinic innovation research and methodological study.

**Pei Sun** is an assistant research fellow of BGI-Beijing Clinical Laboratories, BGI-Shenzhen. She is interested in studying the mechanism of human of genetic disease, pregnancy complications and other pregnancy-related diseases.

**Zicheng Zhao** is a research staff at ByoRyn-Suzhou Life Science and Technology Co., Ltd. His research interests include bioinformatics, machine learning and data analysis.

**Yousheng Yan** is a clinical genetics researcher in Prenatal Diagnostic Center of Beijing Obstetrics and Gynecology Hospital, Capital Medical University. His research interests include novel method of prenatal diagnosis for genetics disease and the pathogenesis of genetic diseases.

**Wentao Yue** is a professor of Central Laboratory in Beijing Obstetrics and Gynecology Hospital at Capital Medical University (Beijing, China). His research interests include birth defect, development and validation of diagnostic marker, and bioinformatics.

**Kai Yang**, PhD, specializes in medical genetics. He focuses on the genetic diagnosis of congenital birth defects and their mechanisms, especially skeletal dysplasia. In the past 5 years, Dr Yang has published 13 articles with a cumulative impact factor of 60.379.

**Ruixia Liu** is a professor and deputy director of the Department of Central Laboratory at Capital Medical University. Her research interests mainly focus on establishing birth cohort and carrying out research on the risk of major birth defects.

**Hui Huang**, PhD, is an associate researcher fellow of BGI Genomics, BGI-Shenzhen. She is also the director of Clinical Research Department, BGI Genomics, BGI-Shenzhen. She is devoted to answering the important questions in clinical science by new biotechnologies and promote the establishment expert standards and guidelines of clinical application of new biotechnology.

**Yinan Wang** is a postdoctoral researcher at Department of Obstetrics and Gynecology of Peking University Shenzhen Hospital. Her research activity focuses on the field of cancer and bioinformatics.

**Yin Chen** is a research staff at ByoRyn-Suzhou Life Science and Technology Co., Ltd. His research interests include machine learning, single cell and platform design.

**Nan Li**, PhD, is an assistant research fellow of BGI Genomics, BGI-Shenzhen. He is a bioinformatics expert of Clinical Research Department, BGI Genomics, BGI-Shenzhen.

**Hailong Feng** is an assistant research fellow of BGI-Beijing Clinical Laboratories, BGI-Shenzhen. His study interests include human of genetic disease, pregnancy complications and other pregnancy-related disease.

**Jing Li** is a research staff at ByoRyn-Suzhou Life Science and Technology Co., Ltd. Her research interests include bioinformatics, data analysis and copy number variations.

**Yifan Liu** is an obstetrician at Prenatal Diagnosis Center, Beijing Obstetrics and Gynecology Hospital, Beijing, China. Her research interests include prenatal diagnosis, fetal medicine and the pathophysiologic mechanisms of infertility for females of advanced age.

**Yujiao Chen**, PhD, is an assistant researcher at Beijing Obstetrics and Gynecology Hospital in China, and graduated from the department of Occupational Health and Environmental Health, School of Public Health, Capital Medical University in Beijing. Her research focuses on the field of environmental pollution-induced birth defects including bioinformatics, data analysis and experiments in biological function.

**Bairong Shen** is a professor at Institutes for Systems Genetics, Frontiers Science Center for Disease-related Molecular Network, West China Hospital, Sichuan University. His research interests include characterization of molecular networks and disease markers, development of new bioinformatics tools and exploration of medical informatics.

**Lijian Zhao**, professor, is the director of BGI Genomics, BGI-Shenzhen. Professor Zhao is committed to the application of next generation sequencing technology to the improvement of human health and the prevention of birth defects.

**Chenghong Yin**, MD, PhD, professor, is the director of Beijing Obstetrics and Gynecology Hospital, Capital Medical University. Professor Yin was committed to basic and clinical research field of infectious diseases, maternal and child health, and birth defects.

## References

[1] McIntyre HD, Catalano P, Zhang C, et al. Gestational diabetes mellitus. Nat Rev Dis Primers 2019;5(1):47. 31296866 10.1038/s41572-019-0098-8

[2] Monteiro LJ, Norman JE, Rice GE, Illanes SE. Fetal programming and gestational diabetes mellitus. Placenta 2016;48:S54–60. 26724985 10.1016/j.placenta.2015.11.015

[3] Pinney SE, Simmons RA. Metabolic programming, epigenetics, and gestational diabetes mellitus. Curr Diab Rep 2012;12:67–74. 22127642 10.1007/s11892-011-0248-1

[4] Alejandro EU, Mamerto TP, Chung G, et al. Gestational diabetes mellitus: a harbinger of the vicious cycle of diabetes. Int J Mol Sci 2020;21(14):5003. 32679915 10.3390/ijms21145003 PMC7404253

[5] Hod M, Kapur A, Sacks DA, et al. The International Federation of Gynecology and Obstetrics (FIGO) initiative on gestational diabetes mellitus: a pragmatic guide for diagnosis, management, and care. Int J Gynaecol Obstet 2015;131:S173–211. 10.1016/S0020-7292(15)30033-3 26433807

[6] Mnatzaganian G, Woodward M, McIntyre HD, et al. Trends in percentages of gestational diabetes mellitus attributable to overweight, obesity, and morbid obesity in regional Victoria: an eight-year population-based panel study. BMC Pregnancy Childbirth 2022;22(1):1–12. 35105311 10.1186/s12884-022-04420-9 PMC8809044

[7] Herring SJ, Oken E. Obesity and diabetes in mothers and their children: can we stop the intergenerational cycle? Curr Diab Rep 2011;11(1):20–7. 20963519 10.1007/s11892-010-0156-9 PMC3191112

[8] Chu SY, Callaghan WM, Kim SY, et al. Maternal obesity and risk of gestational diabetes mellitus. Diabetes Care 2007;30(8):2070–6. 17416786 10.2337/dc06-2559a

[9] Zhao J, Weiler HA. Long-term effects of gestational diabetes on offspring health are more pronounced in skeletal growth than body composition and glucose tolerance. Br J Nutr 2010;104(11):1641–9. 20615268 10.1017/S0007114510002631

[10] Algaba-Chueca F, Maymó-Masip E, Ejarque M, et al. Gestational diabetes impacts fetal precursor cell responses with potential consequences for offspring. Stem Cells Transl Med 2020;9(3):351–63. 31880859 10.1002/sctm.19-0242 PMC7031647

[11] Pathirana MM, Lassi ZS, Ali A, et al. Association between metabolic syndrome and gestational diabetes mellitus in women and their children: a systematic review and meta-analysis. Endocrine 2021;71:310–20. 32930949 10.1007/s12020-020-02492-1

[12] Bhavadharini B, Uma R, Saravanan P, Mohan V. Screening and diagnosis of gestational diabetes mellitus–relevance to low and middle income countries. Clin Diabetes Endocrinol 2016;2(1):1–8. 28702247 10.1186/s40842-016-0031-y PMC5471706

[13] Li-Zhen L, Yun X, Xiao-Dong Z, et al. Evaluation of guidelines on the screening and diagnosis of gestational diabetes mellitus: systematic review. BMJ Open 2019;9(5):e023014. 10.1136/bmjopen-2018-023014 PMC6502228 31061012

[14] Sweeting AN, Ross GP, Hyett J, et al. Gestational diabetes mellitus in early pregnancy: evidence for poor pregnancy outcomes despite treatment. Diabetes Care 2016;39(1):75–81. 26645084 10.2337/dc15-0433

[15] Keshavarz M, Cheung NW, Babaee GR, et al. Gestational diabetes in Iran: incidence, risk factors and pregnancy outcomes. Diabetes Res Clin Pract 2005;69(3):279–86. 16098925 10.1016/j.diabres.2005.01.011

[16] Yang X, Hsu-Hage B, Zhang H, et al. Women with impaired glucose tolerance during pregnancy have significantly poor pregnancy outcomes. Diabetes Care 2002;25(9):1619–24. 12196437 10.2337/diacare.25.9.1619

[17] Kamana KC, Shakya S, Zhang H. Gestational diabetes mellitus and macrosomia: a literature review. Ann Nutr Metab 2015;66(Suppl. 2):14–20. 10.1159/000371628 26045324

[18] Langer O . Fetal macrosomia: etiologic factors. Clin Obstet Gynecol 2000;43(2):283–97. 10863626 10.1097/00003081-200006000-00006

[19] RAN N . Fetal macrosomia in the diabetic patient. Clin Obstet Gynecol 1992;35(1):138–50. 1544239 10.1097/00003081-199203000-00019

[20] Moufarrej MN, Wong RJ, Shaw GM, et al. Investigating pregnancy and its complications using circulating cell-free RNA in women’s blood during gestation. Front Pediatr 2020;8:605219. 33381480 10.3389/fped.2020.605219 PMC7767905

[21] Jiang Y, Zhang Y, Yang Q, et al. The association between fetal fraction and pregnancy-related complications among Chinese population. PloS One 2022;17(7):e0271219. 35819933 10.1371/journal.pone.0271219 PMC9275705

[22] Wu G, Li R, Tong C, et al. Non-invasive prenatal testing reveals copy number variations related to pregnancy complications. Mol Cytogenet 2019;12(1):1–9. 31485271 10.1186/s13039-019-0451-3 PMC6716937

[23] Takahashi H, Yokoi N, Seino S. Glutamate as intracellular and extracellular signals in pancreatic islet functions. Proc Jpn Acad Ser B Phys Biol Sci 2019;95(6):246–60. 10.2183/pjab.95.017 PMC6751295 31189778

[24] Campana WM, Mantuano E, Azmoon P, et al. Ionotropic glutamate receptors activate cell signaling in response to glutamate in Schwann cells. FASEB J 2017;31(4):1744–55. 28073836 10.1096/fj.201601121R PMC5349802

[25] Mao H, Li Q, Gao S. Meta-analysis of the relationship between common type 2 diabetes risk gene variants with gestational diabetes mellitus. PLoS One 2012;7:e45882. 23029294 10.1371/journal.pone.0045882 PMC3454322

[26] Robitaille J, Grant AM. The genetics of gestational diabetes mellitus: evidence for relationship with type 2 diabetes mellitus. Genet Med 2008;10(4):240–50. 18414206 10.1097/GIM.0b013e31816b8710

[27] Li H, Durbin R. Fast and accurate short read alignment with Burrows–Wheeler transform. Bioinformatics 2009;25(14):1754–60. 19451168 10.1093/bioinformatics/btp324 PMC2705234

[28] Broad Institute . “Picard Toolkit.” 2019, GitHub Repository. https://broadinstitute.github.io/picard/.

[29] Danecek P, Bonfield JK, Liddle J, et al. Twelve years of SAMtools and BCFtools. Gigascience 2021;10(2):giab008. 33590861 10.1093/gigascience/giab008 PMC7931819

[30] Chen L, Qing Y, Li R, et al. Somatic variant analysis suite: copy number variation clonal visualization online platform for large-scale single-cell genomics. Brief Bioinform 2022;23(1):bbab452. 10.1093/bib/bbab452 34671807

[31] Wang RH, Wang J, Li SC. Probabilistic tensor decomposition extracts better latent embeddings from single-cell multiomic data. Nucleic Acids Res 2023;51(15):e81. 10.1093/nar/gkad570 PMC10450184 37403780

[32] Abadi M, Barham P, Chen J, et al. Tensorflow: a system for large-scale machine learning. Osdi 2016;2016(16):265–83.

[33] Pedregosa F, Varoquaux G, Gramfort A, et al. Scikit-learn: machine learning in python. J Mach Learn Res 2011;12:2825–30.

[34] Adalsteinsson VA, Ha G, Freeman SS, et al. Scalable whole-exome sequencing of cell-free DNA reveals high concordance with metastatic tumors. Nat Commun 2017;8(1):1324. 29109393 10.1038/s41467-017-00965-y PMC5673918

[35] Panzer JK, Tamayo A, Caicedo A. Restoring glutamate receptor signaling in pancreatic alpha cells rescues glucagon responses in type 1 diabetes. Cell Rep 2022;41(11):111792. 36516761 10.1016/j.celrep.2022.111792

[36] Dai XQ, Camunas-Soler J, Briant LJB, et al. Heterogenous impairment of α cell function in type 2 diabetes is linked to cell maturation state. Cell Metab 2022;34(2):256, e5–268.e5. 35108513 10.1016/j.cmet.2021.12.021 PMC8852281

[37] Abarkan M, Gaitan J, Lebreton F, et al. The glutamate receptor GluK2 contributes to the regulation of glucose homeostasis and its deterioration during aging. Mol Metab 2019;30:152–60. 31767166 10.1016/j.molmet.2019.09.011 PMC6807305

[38] Wachsmuth HR, Weninger SN, Duca FA. Role of the gut–brain axis in energy and glucose metabolism. Exp Mol Med 2022;54(4):377–92. 35474341 10.1038/s12276-021-00677-w PMC9076644

[39] Gendaszewska-Darmach E, Garstka MA, Błażewska KM. Targeting small GTPases and their prenylation in diabetes mellitus. J Med Chem 2021;64(14):9677–710. 34236862 10.1021/acs.jmedchem.1c00410 PMC8389838

[40] Collares-Buzato CB, Carvalho CP. Is type 2 diabetes mellitus another intercellular junction-related disorder? Exp Biol Med (Maywood) 2022;247(9):743–55. 35466731 10.1177/15353702221090464 PMC9134762

[41] Zhong H, Duan BH, Du FM, et al. Identification of key genes, biological functions, and pathways of empagliflozin by network pharmacology and its significance in the treatment of type 2 diabetes mellitus. Ann Transl Med 2023;11(2):123. 36819540 10.21037/atm-22-6406 PMC9929817

[42] Bryk-Wiązania AH, Undas A. Hypofibrinolysis in type 2 diabetes and its clinical implications: from mechanisms to pharmacological modulation. Cardiovasc Diabetol 2021;20(1):191. 34551784 10.1186/s12933-021-01372-w PMC8459566

