# Supplementary figures and images for: Identify gestational diabetes mellitus by deep learning model from cell-free DNA at the early gestation stage
Source: Brief Bioinform. 2024 Jan 2;25(1):bbad492. doi: 10.1093/bib/bbad492 (PMC10782912; doi:10.1093/bib/bbad492)

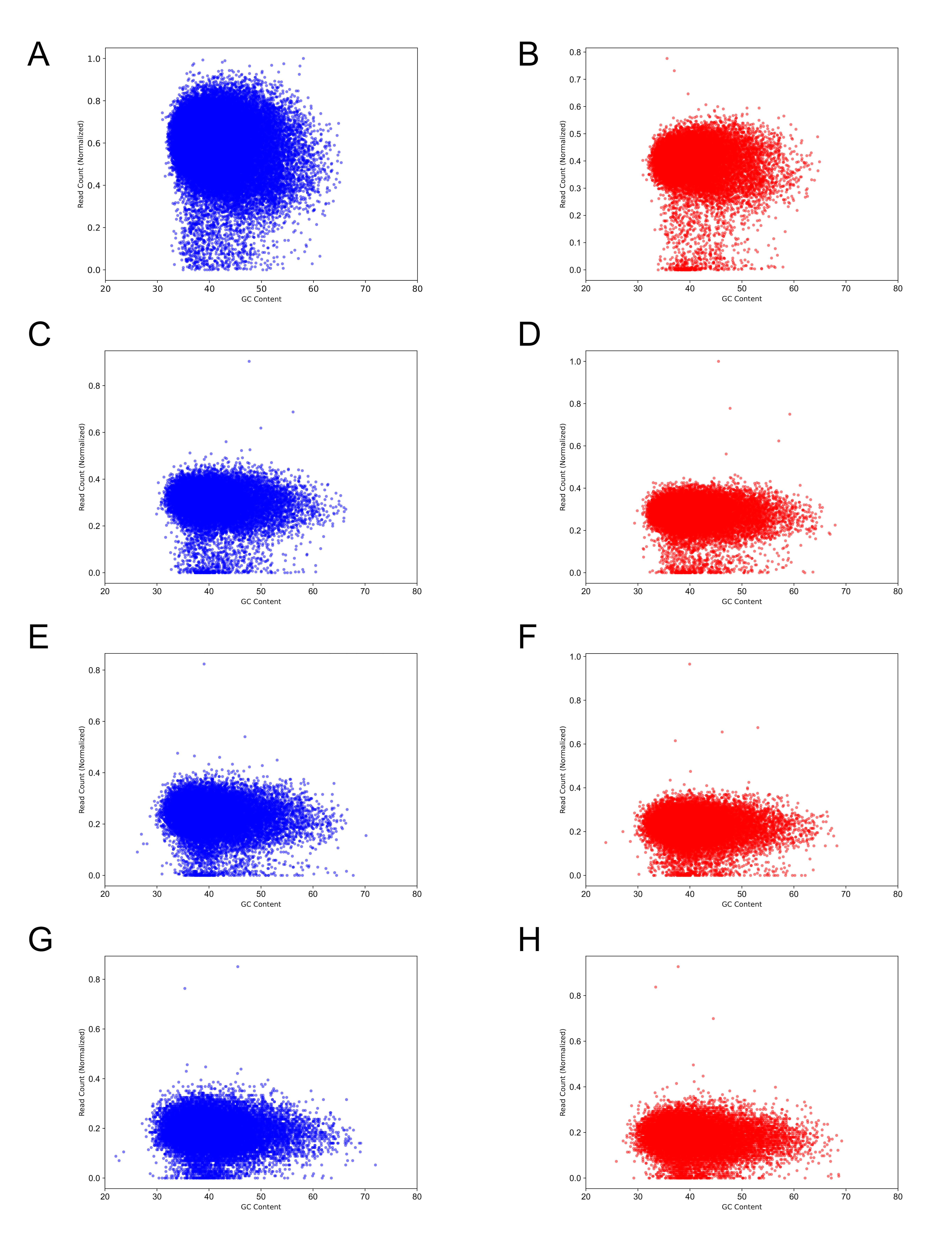

Supplement: Supplementary_material_bbad492 [file supplementary_material_bbad492.zip › Supplementary Figure 2.tiff]

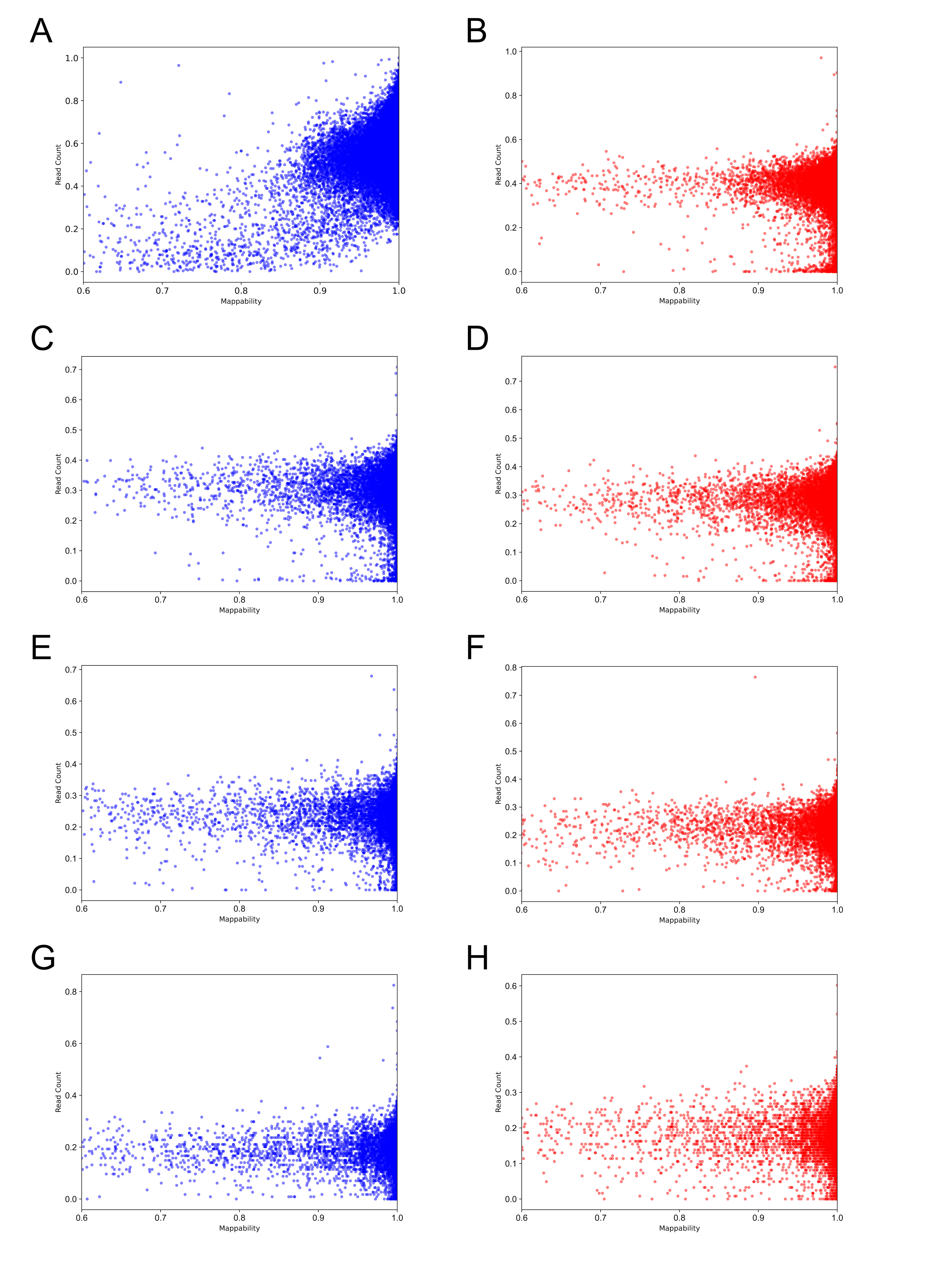

Supplement: Supplementary_material_bbad492 [file supplementary_material_bbad492.zip › Supplementary Figure 3.tiff]

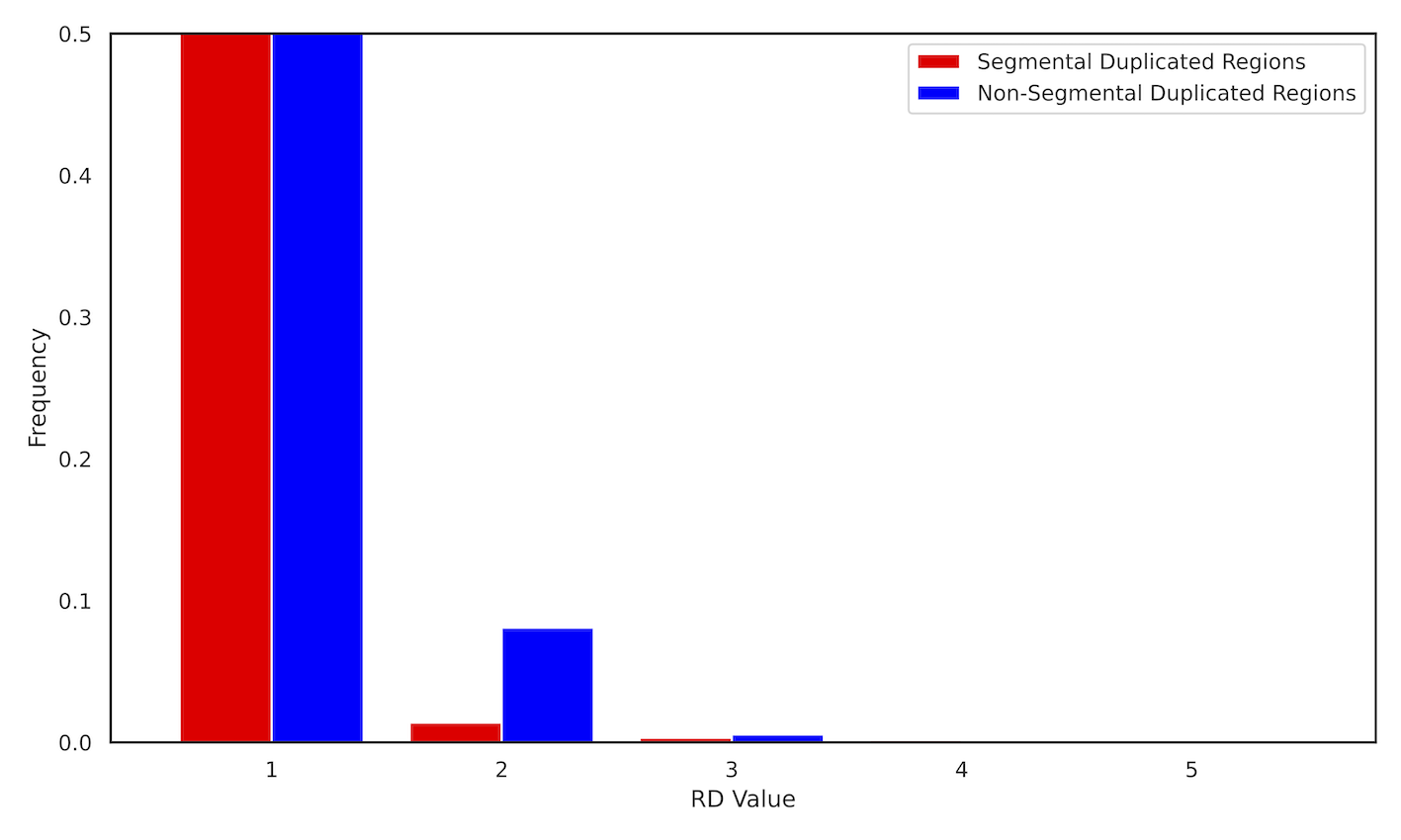

Supplement: Supplementary_material_bbad492 [file supplementary_material_bbad492.zip › Supplementary_Figure_1.tiff]

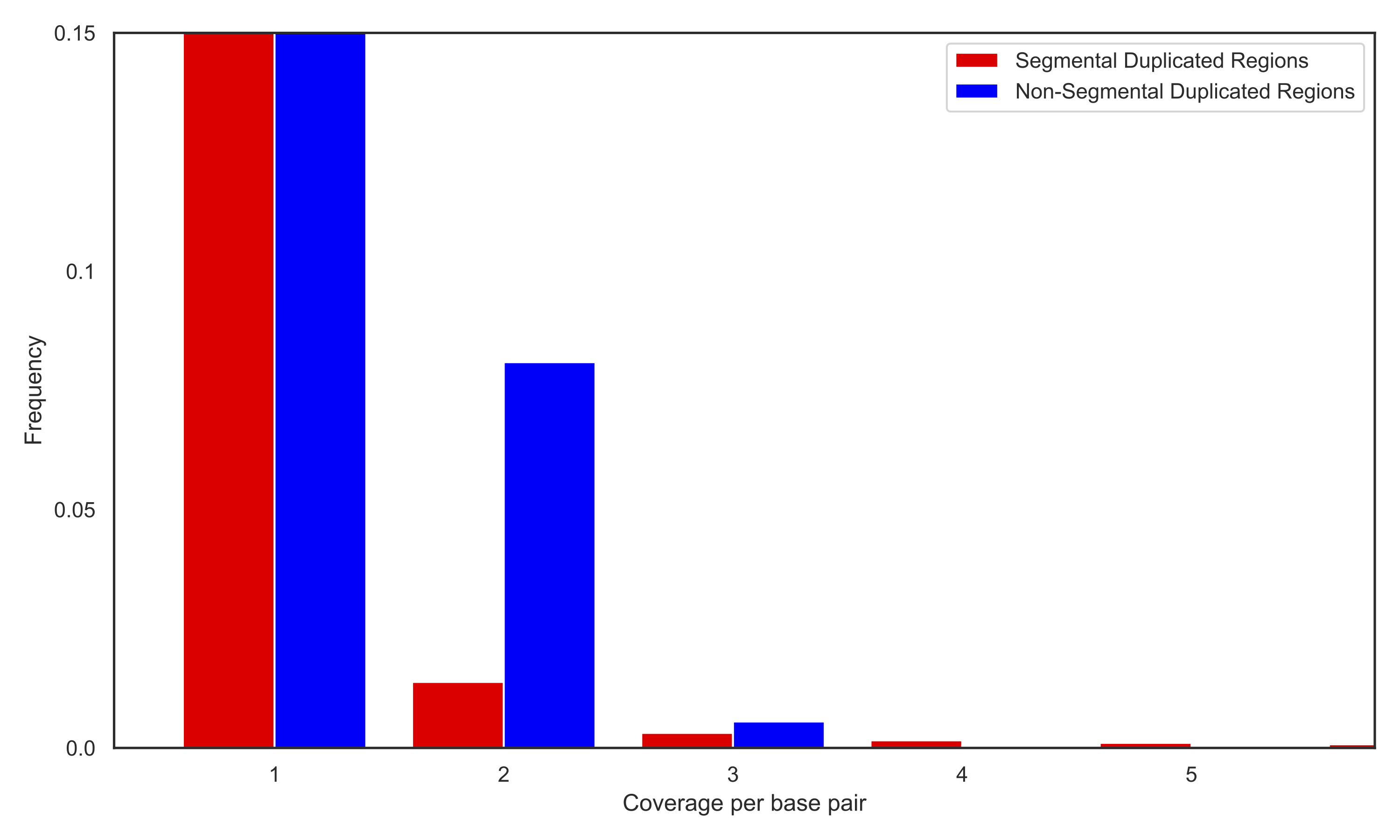

Supplement: Supplementary_material_bbad492 [file supplementary_material_bbad492.zip › Supplementary_Figure_4.tiff]

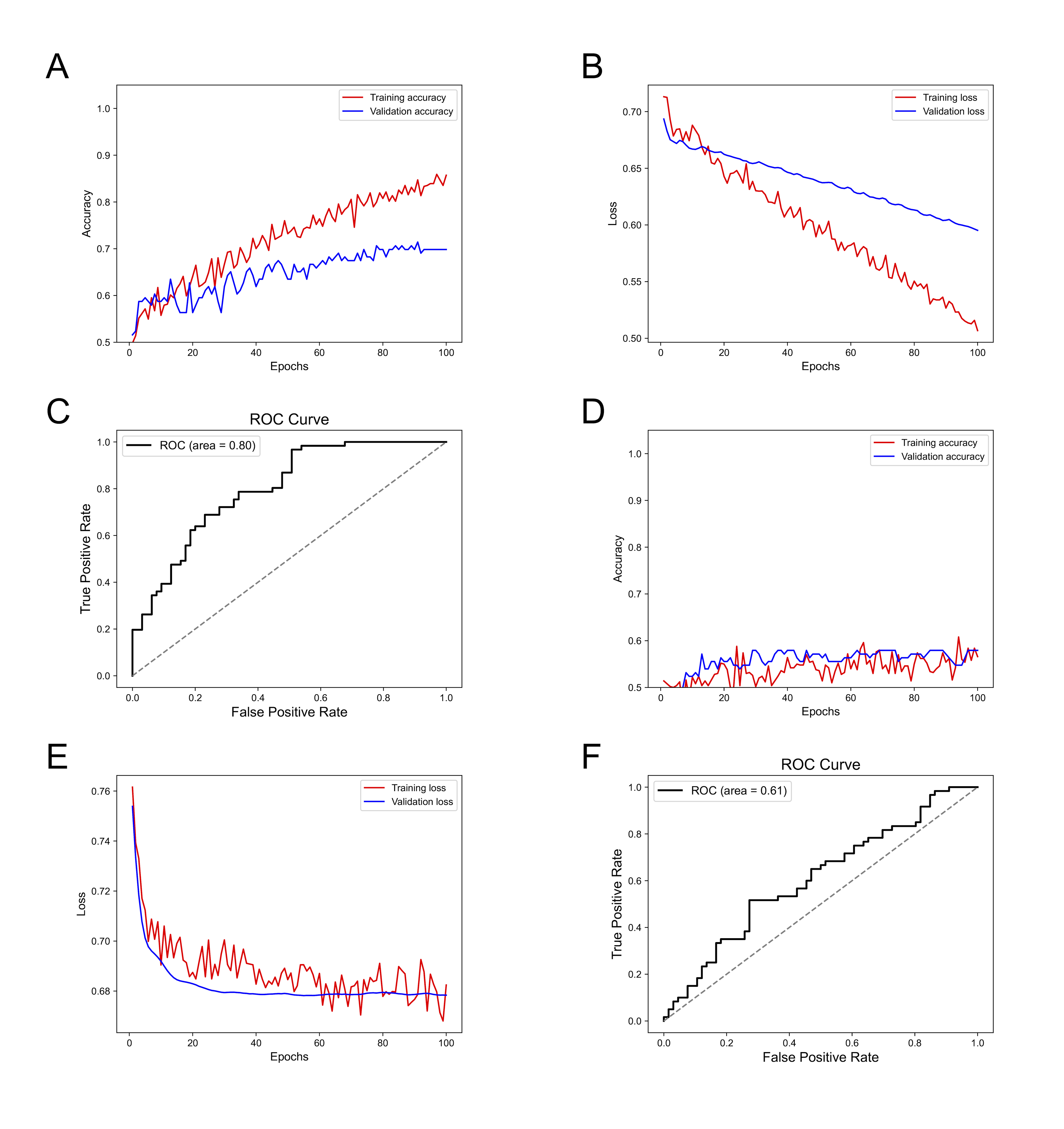

Supplement: Supplementary_material_bbad492 [file supplementary_material_bbad492.zip › Supplementary_Figure_5.tiff]
